# Nonlocal Total Variation Using the First and Second Order Derivatives and Its Application to CT image Reconstruction

**DOI:** 10.3390/s20123494

**Published:** 2020-06-20

**Authors:** Yongchae Kim, Hiroyuki Kudo

**Affiliations:** Department of Computer Science, Graduate School of Systems and Information Engineering, University of Tsukuba, Tennoudai 1-1-1, Tsukuba 305-8573, Japan; acevip12@hotmail.com

**Keywords:** image reconstruction, computed tomography, compressed sensing, nonlocal total variation, sparse-view CT, low-dose CT, proximal splitting, row-action, brain CT image

## Abstract

We propose a new class of nonlocal Total Variation (TV), in which the first derivative and the second derivative are mixed. Since most existing TV considers only the first-order derivative, it suffers from problems such as staircase artifacts and loss in smooth intensity changes for textures and low-contrast objects, which is a major limitation in improving image quality. The proposed nonlocal TV combines the first and second order derivatives to preserve smooth intensity changes well. Furthermore, to accelerate the iterative algorithm to minimize the cost function using the proposed nonlocal TV, we propose a proximal splitting based on Passty’s framework. We demonstrate that the proposed nonlocal TV method achieves adequate image quality both in sparse-view CT and low-dose CT, through simulation studies using a brain CT image with a very narrow contrast range for which it is rather difficult to preserve smooth intensity changes.

## 1. Introduction

Nonlocal Total Variation (TV) [1,2,3,4,5,6] was proposed as an improved version of ordinary TV. Nonlocal TV can use a weighting function (e.g., the weight of nonlocal means filter) by taking the intensity difference between the pixel pair into account, and can obtain higher image quality than the ordinary TV that uses only pairs of adjacent pixels.

Since G. Gilboa and S. Osher (2009) [5] proposed nonlocal operator, nonlocal TV has been widely applied to image reconstruction problems in sparse-view CT and low-dose CT [1,2,3,4].

H. Kim et al. (2016) [2] applied nonlocal TV to sparse-view CT and showed that nonlocal TV improves image quality over ordinary TV and incorporating the reweighted L1 norm into nonlocal TV further improves tissue contrast and structural details. Following this, K. Kim et al. (2017) [3] applied nonlocal TV to low-dose CT and showed nonlocal TV is effective for low-dose noise (Poisson noise).

D. Lv et al. (2019) [4] proposed a hybrid prior distribution (called NLTG prior) that combines nonlocal TV with Gaussian distribution. Additionally, they showed the possibility of applying NLTG prior to a large class of image reconstruction problems, especially when reference images were available.

However, the most existing nonlocal TV studies [2,3,4] are based on the first-order derivative, and still contain the staircase artifact problem as a potential drawback. Since the first-order derivative is too sensitive to the pixel values, even linear intensity changes are detected as false edges, which leads to staircase artifacts in the same way as local TV does [7,8,9].

On the other hand, higher-order derivatives (the second-order or more) possesses a potential risk that, as the order of differentiation is larger, its ability enhance image edges is smaller leading to an image blurring problem.

In previous studies, to overcome the disadvantage of using only the first-order or only the second-order, Bredies et al. (2010) [10] proposed Total Generalized Variation (TGV) that involves and balances higher-order derivatives and showed impressive results in the denoising problem. After that work, Ranftl et al. (2014) [11] proposed a nonlocal version of TGV.

Our proposed method is also a combined method of the first and second order derivatives. We define the regularization term as a weighted sum of two terms, where the first term is the ordinary nonlocal TV based on the first-order and the second term is based on the second-order. Additionally, in this paper, we introduce a newly discovered idea called nonlocal Total K-Split Variation, which is based on the second-order derivative using nonlocal regularization. This idea allows us to preserve smooth intensity changes well.

Furthermore, to accelerate the iterative algorithm associated with the proposed nonlocal TV, we propose a specially designed proximal splitting based on Passty’s framework. In this proximal splitting, as the number of dividing the cost function into small subfunctions increases, the faster convergence is achieved [12,13,14]. The structure of final iterative algorithm is row-action type with respect to both the data-fidelity term and the regularization term [15,16,17], which converges to a minimizer very quickly [18,19,20,21,22].

Finally, in order to demonstrate the performances of combining the first and second order derivatives in reconstructed images, we use a brain CT image, in which a very narrow contrast range is used to display the image, where it is very difficult to preserve smooth intensity changes. Also, simulation studies are performed for both the sparse-view CT and the low-dose CT. We demonstrate that the proposed nonlocal TV method achieves adequate image quality within a small number of iterations.

## 2. Methodology

### 2.1. Problem Definition

We define the following unconstrained cost function:

(1) $$\underset{\vec{x}\geq 0}{argmin}J\left(\vec{x}\right)=f\left(\vec{x}\right)+u\left(\vec{x}\right)=\|A\vec{x}-{\vec{b}\|}_{2}^{2}+\beta \omega \|W{\vec{x}\|}_{1}^{1},$$

where $f\left(\vec{x}\right)=\|A\vec{x}-{\vec{b}\|}_{2}^{2}$ is the data-fidelity term, and $u\left(\vec{x}\right)=\beta \omega \|W{\vec{x}\|}_{1}^{1}$ is the regularization term, and $A=\left\{a_{ij}\right\}$ is the I×J system matrix, β is the hyper-parameter to control regularization strength, and ω is the weight of regularization term, and W is the sparsifying transform to make $W\vec{x}$ sparse. Image reconstruction is an inverse problem to recover the image of attenuation coefficients $\vec{x}={\left(x_{1},x_{2},\ldots ,x_{J}\right)}^{T}$ from the measured projection data $\vec{b}={\left(b_{1},b_{2},\ldots ,b_{I}\right)}^{T}$.

In the sparse-view CT [23,24], by using the projection data corresponding to less than 100 directions (the conventional CT uses 1000–2000 projection data), the equation $A\vec{x}=\vec{b}$ becomes severely underdetermined, i.e., the dimension J of unknowns $\vec{x}$ is larger than the dimension I of measurements $\vec{b}$ (I<J). In this case, the regularization term acts to avoid the ill-posed problem by introducing the prior knowledge that most components of the vector $W\vec{x}$ are close to 0.

On the other hand, in the low-dose CT, the equation $A\vec{x}=\vec{b}$ becomes inconsistent due to Poisson noise $\vec{n}$ ($A\vec{x}-\vec{b}=\vec{n}$). In this case, the regularization term helps to reduce the effect of Poisson noise $\vec{n}$ by a smoothing.

First, we begin by the modified anisotropic nonlocal TV based on the first-order derivative expressed as

(2) $$Nonlocal\;TV=u^{TV}\left(\vec{x}\right)=\beta t\overset{J}{\underset{j=1}{\sum }}\underset{j^{\prime }\in \Omega }{\sum }\omega_{jj^{\prime }}\left|x_{j}-x_{j^{\prime }}\right|,$$

where $x_{j}$ is the intensity in the pixel j, and $x_{j^{\prime }}$ is the intensity in a distant pixel $j^{\prime }$, and $\omega_{jj^{\prime }}$ is the weight of smoothing assigned for each pixel pair $\left(x_{j},x_{j^{\prime }}\right)$.

**Figure 1:**
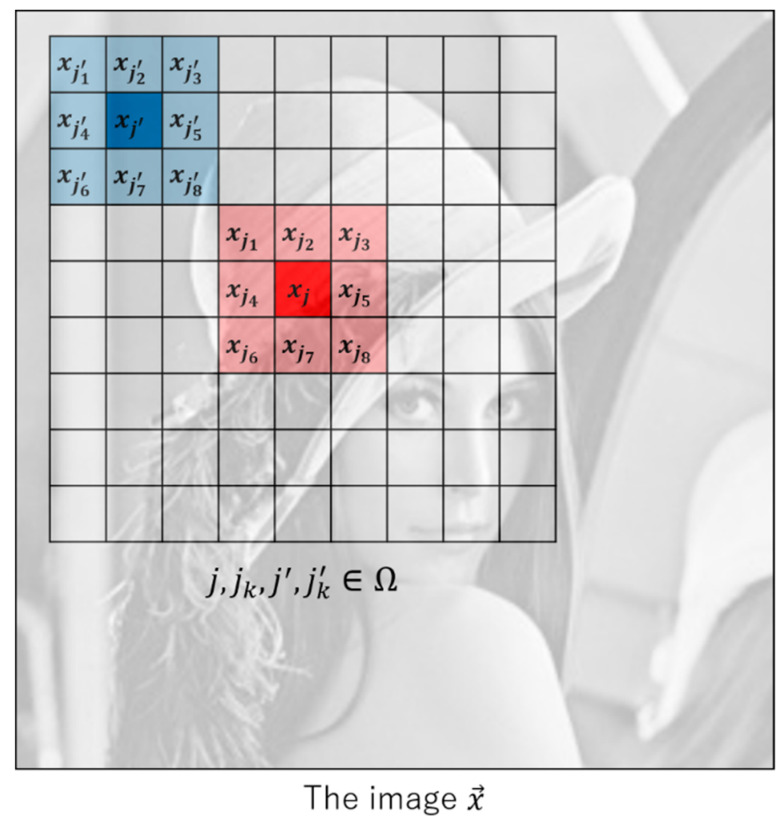
Definition of the pixel location in the proposed regularization term corresponding to k=1, 2, 3,⋯, 7, 8.

Next, we describe a newly discovered regularization term based on the second-order derivative called nonlocal Total K-split Variation (TKV). The main idea is to consider more derivatives around $x_{j}$ and $x_{j^{\prime }}$ as

(3) $$Nonlocal\;TKV=u^{TKV}\left(\vec{x}\right)=\frac{\beta \left(1-t\right)}{8}\overset{J}{\underset{j=1}{\sum }}\underset{j^{\prime }\in \Omega }{\sum }\overset{8}{\underset{k=1}{\sum }}\omega_{jj^{\prime }}\left|\left(x_{j}-x_{j_{k}}\right)-\left(x_{j^{\prime }}-x_{j_{k}^{\prime }}\right)\right|,$$

where $x_{j_{k}}$ is the adjacent pixel value of $x_{j}$, $x_{j_{k}^{\prime }}$ is the adjacent pixel value of $x_{j^{\prime }}$. We remark that the TKV term is divided into a sum of eight terms dependent of the direction to take the pixel difference as shown in Figure 1.

In the proposed method, we assume that the reconstructed image $\vec{x}$ is close to piecewise-polynomial of first order. Under this assumption, our proposed regularization term is designed to include both the first and second order derivatives as

(4) $$u\left(\vec{x}\right)=\beta \overset{J}{\underset{j=1}{\sum }}\underset{j^{\prime }\in \Omega }{\sum }\omega_{jj^{\prime }}\left[t\left|x_{j}-x_{j^{\prime }}\right|+\frac{\left(1-t\right)}{8}\overset{8}{\underset{k=1}{\sum }}\left|\left(x_{j}-x_{j_{k}}\right)-\left(x_{j^{\prime }}-x_{j_{k}^{\prime }}\right)\right|\right],\left(0<t<1\right),$$

where the proposed regularization term is a combination of two terms ($u\left(\vec{x}\right)=u^{TV}\left(\vec{x}\right)+u^{TKV}\left(\vec{x}\right)$). Additionally t is the trade-off parameter between nonlocal TV and TKV. If t is large, the reconstructed image becomes closer to that of nonlocal TV. If t is small, the reconstructed image becomes closer to that of nonlocal TKV. To match the strength of the first term into that of the second term, we divide the second-order derivative based nonlocal TKV into 8 directions. Figure 1 shows the location of pixels appearing in $u^{TV}\left(\vec{x}\right)$ and $u^{TKV}\left(\vec{x}\right)$ for k=1, 2, 3,⋯, 7, 8.

### 2.2. Accelerated Algorithm Using the Proximal Splitting with Passty’s Framework

To accelerate the iterative algorithm to minimize the cost function, we propose a specially designed proximal splitting with Passty’s framework. The ordinary proximal splitting is able to minimize a cost function consisting of a sum of only two component terms, where the proximity operator corresponding to each subfunction is applied alternately [12,13]. On the other hand, by using Passty’s framework which is not well-known in the image reconstruction community, a cost function can be divided into a number of much simpler subfunctions [14]. This results in considerable benefits for optimization problems appearing in CT reconstruction. By applying the proximity operator corresponding to each subfunction alternately, the proposed algorithm possesses a form of row-action type [15,16,17], which converges to a minimizer very quickly [18,19,20,21,22].

We begin by a brief review the proximal splitting with Passty’s framework. Let us consider a convex minimization problem formulated as

(5) $$\underset{\vec{x}}{\mathrm{argmin}}J\left(\vec{x}\right),$$

where $J\left(\vec{x}\right)$ is a lower semi-continuous (lsc) convex function.

The proximity operator corresponding to the function $J\left(\vec{x}\right)$ is defined as

(6) $$\vec{x}=prox_{\alpha J}\left(\vec{z}\right)\equiv \underset{\vec{x}}{\mathrm{argmin}}\left(J\left(\vec{x}\right)+\frac{1}{2\alpha }\|\vec{x}-{\vec{z}\|}_{2}^{2}\right),$$

where α is the parameter called step-size. We note that $J\left(\vec{x}\right)$ can be a non-differentiable function such as component terms of TV or nonlocal TV. The proximity operator is a non-expansive mapping such that its fixed-points $\vec{x}$ satisfying $\vec{x}=prox_{\alpha J}\left(\vec{x}\right)$ coincides with a minimizer of $J\left(\vec{x}\right)$ for any α>0. Therefore, the minimization problem of $J\left(\vec{x}\right)$ can be solved by the iterative formula expressed as ${\vec{x}}^{\left(n+1\right)}=prox_{\alpha J}\left({\vec{x}}^{\left(n\right)}\right)$ (i.e., proximal algorithm). Next, we are going to explain the proximal splitting with Passty’s framework.

**[Passty’s framework]** Let us consider the case where $J\left(\vec{x}\right)$ can be divided into a sum of subfunctions as:

(7) $$J\left(\vec{x}\right)=\overset{I}{\underset{i=1}{\sum }}J_{i}\left(\vec{x}\right).$$

The iterative algorithm can be constructed by applying the proximity operator corresponding to each subfunction $J_{i}\left(\vec{x}\right)\;\left(i=1,2,\ldots ,I\right)$ as below

(8) $${\vec{x}}^{\left(n+1\right)}=prox_{\alpha^{\left(n\right)}J_{I}}\cdot \cdots \cdot prox_{\alpha^{\left(n\right)}J_{2}}\cdot prox_{\alpha^{\left(n\right)}J_{1}}\left({\vec{x}}^{\left(n\right)}\right).$$

Furthermore, let us consider the case where $J_{i}\left(\vec{x}\right)$ is a sum of two subfunctions, like our cost function including data-fidelity term and regularization term as

(9) $$J_{i}\left(\vec{x}\right)=f_{i}\left(\vec{x}\right)+u\left(\vec{x}\right).$$

The update can be constructed by applying two operators corresponding to each subfunction alternately as below

(10) $${\vec{x}}^{\left(n+1\right)}=prox_{\alpha^{\left(n\right)}u}\cdot prox_{\alpha^{\left(n\right)}f_{i}}\left({\vec{x}}^{\left(n\right)}\right).$$

Finally, we show in Algorithm 1 the optimization model applied to this paper:

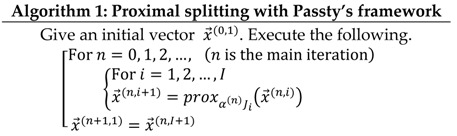


In Passty’s framework, by increasing the number to divide the cost function into smaller subfunctions, the better convergence can be expected. In this paper, this division is performed as follows. First, the data-fidelity term is divided as shown in Equation (7) in such a way that each subfunction $f_{i}\left(\vec{x}\right)$ contains only a single term corresponding to projection data $b_{i}$. Therefore, the final algorithm can be designed in the form of a row-action type algorithm such as ART method.

We mention that $u\left(\vec{x}\right)$ can also be divided into a sum of many subfunctions. In this paper, we divide $u\left(\vec{x}\right)$ as finely as possible similarly to the case of the data-fidelity term. This idea leads to a significant benefit to simplify the processing of nonlocal TV+TKV term as well as improving the convergence speed. With respect to the regularization term $u\left(\vec{x}\right)$, we perform a division described in Section 2.3.2.

### 2.3. Optimization

In this section, we focus on how to divide the cost function and how to derive the resulting iterative algorithm.

#### 2.3.1. Update the Data-Fidelity Term

The data-fidelity term can be divided into I subfunctions as below

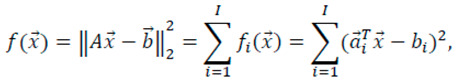
(11)
where I is the number of projection data, and ${\vec{a}}_{i}$ is *i*-th row vector of the system matrix A, and $b_{i}$ is *i*-th component of projection data. Furthermore, we note that $f_{i}\left(\vec{x}\right)$ is a subfunction corresponding to the data-fidelity term.

The minimization problem for the data-fidelity term can be defined as

(12) $${\vec{x}}^{\left(n,i+1\right)}=prox_{\alpha^{\left(n\right)}f_{i}}\left({\vec{x}}^{\left(n,i\right)}\right)=\underset{\vec{x}}{\mathrm{argmin}}\left\{{\left({\vec{a}}_{i}^{T}\vec{x}-b_{i}\right)}^{2}+\frac{1}{2\alpha^{\left(n\right)}}\|\vec{x}-{\vec{x}}^{\left(n,i\right)}\|{}_{2}^{2}\right\}.$$

By introducing a slack variable 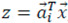, the above minimization problem for each subfunction $f_{i}\left(\vec{x}\right)$ can be converted into the constrained minimization below

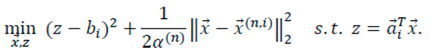
(13)

The Lagrange function can be defined by

(14) $$L_{f_{i}}\left(\vec{x},z,\lambda \right)={\left(z-b_{i}\right)}^{2}+\frac{1}{2\alpha^{\left(n\right)}}\|\vec{x}-{\vec{x}}^{\left(n,i\right)}\|{}_{2}^{2}+\lambda \left(z-{\vec{a}}_{i}^{T}\vec{x}\right),$$

where λ is the Lagrange multiplier called the dual variable.

Finally, by optimizing the Lagrange function, we obtain the following expression of row-action type iteration.

(15) $${\vec{x}}^{\left(n,i+1\right)}={\vec{x}}^{\left(n,i\right)}+\alpha^{\left(n\right)}\frac{b_{i}-{\vec{a}}_{i}^{T}{\vec{x}}^{\left(n,i\right)}}{1/2+\alpha^{\left(n\right)}\|{\vec{a}}_{i}\|{}_{2}^{2}}{\vec{a}}_{i},\;\alpha^{\left(n\right)}=\frac{\alpha_{0}}{1+\varepsilon n},\left(i\in R_{\left[I\right]}\right),\;(\alpha_{0}:\;\mathrm{initial}\;\mathrm{value}\;\mathrm{of}\;\mathrm{step}\;\mathrm{size},\;\varepsilon :\;\mathrm{deceleration}\;\mathrm{rate}\;\mathrm{of}\;\mathrm{step}\text{-}\mathrm{size},\;R_{\left[I\right]}:\;\mathrm{ordered}\;\mathrm{subsets})$$

where $\alpha^{\left(n\right)}$ is the step-size parameter to control the convergence, and n is the number of main iterations. After updating all elements of projection data, n is increased by 1. The step-size $\alpha^{\left(n\right)}$ is diminished gradually to zero as the iteration proceeds (i.e. diminishing step-size rule). In Passty’s framework, it is known that this diminishing contributes to ensuring an exact convergence to a minimizer, thereby, avoiding the so-called limit cycle problem. Furthermore, we introduce a random-access order of projection data $R_{\left[I\right]}$ to enable a fast convergence within 20–30 iterations [19,20]. The mathematical detail to derive the update formula in Equation (15) has been already described in our previous studies [19,20,21,22].

#### 2.3.2. Update the Regularization Term

We describe how to divide the regularization term. The modified anisotropic nonlocal TV possesses the following structure and is called L1 based group LASSO here. The L1 based group LASSO possesses a very simple structure, where each absolute value term can be considered a group element.

(16) $$u^{TV}\left(\vec{x}\right)=\beta t\overset{J}{\underset{j=1}{\sum }}\underset{j^{\prime }\in \Omega }{\sum }\omega_{jj^{\prime }}\left|x_{j}-x_{j^{\prime }}\right|=\beta t\overset{G}{\underset{g=1}{\sum }}{\left[\omega_{jj^{\prime }}\|{\vec{x}}_{j}-{\vec{x}}_{j^{\prime }}\|{}_{1}^{1}\right]}_{g}=\beta t\overset{G}{\underset{g=1}{\sum }}u_{g}^{TV}\left(\vec{x}\right),\;\left(j=g\right).$$

where g is the group index, and the group index g corresponds to the pixel index j, and the TV term can be divided into G groups (G subfunctions). Furthermore, the group itself becomes a subfunction $u_{g}^{TV}\left(\vec{x}\right)$.

In the case of the TKV term, it can be divided into (G×8) subfunctions as below

(17) $$u^{TKV}\left(\vec{x}\right)=\frac{\beta \left(1-t\right)}{8}\overset{8}{\underset{k=1}{\sum }}\overset{G}{\underset{g=1}{\sum }}{\left[\omega_{jj^{\prime }}\|\left({\vec{x}}_{j}-{\vec{x}}_{j_{k}}\right)-{\left({\vec{x}}_{j^{\prime }}-{\vec{x}}_{j_{k}^{\prime }}\right)\|}_{1}^{1}\right]}_{g}=\frac{\beta \left(1-t\right)}{8}\overset{8}{\underset{k=1}{\sum }}\overset{G}{\underset{g=1}{\sum }}u_{g}^{TKV}\left(\vec{x}\right).$$

**Figure 2:**
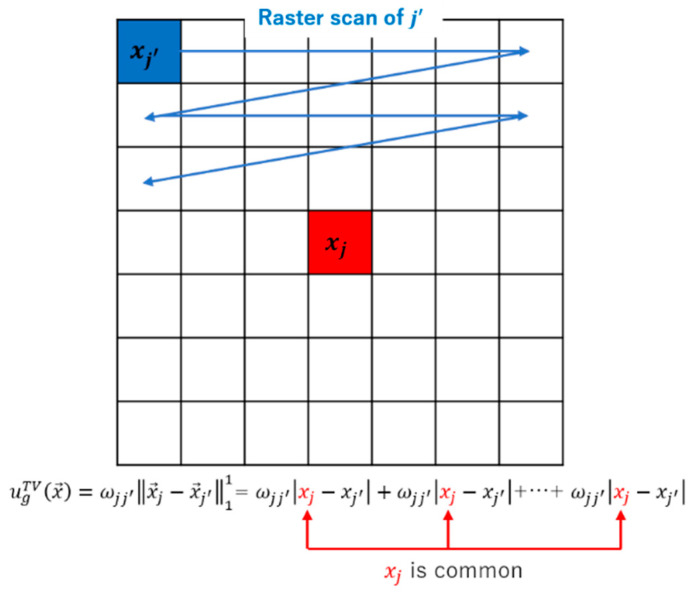
Raster scanning during the update ($j^{\prime }=1,\;2,\;3,\;\ldots \;$).

The detailed structure of the TV term is shown in Figure 2. Among the elements of the group, the pixel j is common and distant pixel $j^{\prime }$ has different values for each other.

The sequential update is related to raster scanning. 

In the TV term, when assuming that $x_{j}$ and $x_{j^{\prime }}$ are updated simultaneously, $x_{j}$ is updated sequentially $J^{\prime }$ times, and $x_{j^{\prime }}$ is updated once respectively. 

In the case of TKV term, when assuming that $x_{j}$, $x_{j_{k}}$, $x_{j^{\prime }}$, $x_{j_{k}^{\prime }}$ are updated simultaneously, $x_{j}$ is updated sequentially $\left(J^{\prime }\times 8\right)$ times, and $x_{j^{\prime }}$ is updated sequentially eight times. Following this, $x_{j_{k}}$ and $x_{j_{k}^{\prime }}$ (the adjacent pixels) are updated once respectively.

**[Update the TV term]** First, we consider updating the pixel j.

The proximity operator for a subfunction can be defined as follows

(18) $$\underset{{\vec{x}}_{j}}{\mathrm{argmin}}\left(\omega_{jj^{\prime }}\|{\vec{x}}_{j}-{\vec{x}}_{j^{\prime }}^{\left(n\right)}\|{}_{1}^{1}+\frac{1}{2\alpha^{\left(n\right)}}\|{\vec{x}}_{j}-{\vec{x}}_{j}^{\left(n,j^{\prime }\right)}\|{}_{2}^{2}\right),$$

where $x_{j^{\prime }}^{\left(n\right)}$ is the current updated solution as a constant approximation, which is the image updated from the data-fidelity term. The above L1 norm minimization problems can be solved with the following soft-thresholding.

(19) $$S_{\nabla }\left(x_{j}^{\left(n,j^{\prime }\right)}\right)=x_{j}^{\left(n,j^{\prime }\right)}-\{\begin{array}{c} \nabla ,\;\tau >\nabla \\ -\nabla ,\;\tau <-\nabla \\ \;\tau_{TV}\;\left(otherwise\right) \end{array},\;\left(\tau_{TV}=\left(x_{j}^{\left(n,j^{\prime }\right)}-x_{j^{\prime }}^{\left(n\right)}\right),\nabla =\alpha^{\left(n\right)}\beta t\omega_{jj{}^{\prime }}\right),$$

where $S_{\nabla }\left(x_{j}^{\left(n,j^{\prime }\right)}\right)$ is the soft-thresholding function. The solution ($S_{\nabla }\left(x_{j}^{\left(n,j^{\prime }\right)}\right)$) corresponding to otherwise is simply $x_{j^{\prime }}^{\left(n\right)}$.

For better convergence, although it is possible to update only pixel j, we update the pixel j and $j^{\prime }$ simultaneously.

For updating the pixel j and $j^{\prime }$, we further divide a subfunction $u_{g}^{TV}\left(\vec{x}\right)$ into two subfunctions as below

(20) $$u^{TV}\left(\vec{x}\right)=\beta t\overset{G}{\underset{g=1}{\sum }}u_{g}^{TV}\left(\vec{x}\right)=\beta t\overset{G}{\underset{g=1}{\sum }}{\left[\omega_{jj{}^{\prime }}\|{\vec{x}}_{j}-{\vec{x}}_{j^{\prime }}\|{}_{1}^{1}\right]}_{g}\;\;=\beta t\overset{G}{\underset{g=1}{\sum }}{\left[{\left[\omega_{jj^{\prime }}\|{\frac{{\vec{x}}_{j}-{\vec{x}}_{j^{\prime }}}{2}\|}_{1}^{1}\right]}_{x_{j}}+{\left[\omega_{jj^{\prime }}\|{\frac{{\vec{x}}_{j}-{\vec{x}}_{j^{\prime }}}{2}\|}_{1}^{1}\right]}_{x_{j^{\prime }}}\right]}_{g}\;\;=\beta t\overset{G}{\underset{g=1}{\sum }}{\left[p\left({\vec{x}}_{j}\right)+p\left({\vec{x}}_{j^{\prime }}\right)\right]}_{g}.$$

where $p\left({\vec{x}}_{j}\right)={\left[\;\right]}_{x_{j}}$, $p\left({\vec{x}}_{j^{\prime }}\right)={\left[\;\right]}_{x_{j^{\prime }}}$.

The proximity operator for each subfunction ($prox_{\alpha^{\left(n\right)}p}\left({\vec{x}}_{j}^{\left(n,j^{\prime }\right)}\right)$, $prox_{\alpha^{\left(n\right)}p}\left({\vec{x}}_{j^{\prime }}^{\left(n\right)}\right)$) can be defined as follows

(21) $$\underset{{\vec{x}}_{j}}{\mathrm{argmin}}\left(\omega_{jj^{\prime }}\|{\vec{x}}_{j}-{\frac{{\vec{x}}_{j}^{\left(n,j^{\prime }\right)}+{\vec{x}}_{j^{\prime }}^{\left(n\right)}}{2}\|}_{1}^{1}+\frac{1}{2\alpha^{\left(n\right)}}\|{\vec{x}}_{j}-{\vec{x}}_{j}^{\left(n,j^{\prime }\right)}\|{}_{2}^{2}\right),\;\underset{{\vec{x}}_{j^{\prime }}}{\mathrm{argmin}}\left(\omega_{jj^{\prime }}\|-{\vec{x}}_{j^{\prime }}+{\frac{{\vec{x}}_{j}^{\left(n,j^{\prime }\right)}+{\vec{x}}_{j^{\prime }}^{\left(n\right)}}{2}\|}_{1}^{1}+\frac{1}{2\alpha^{\left(n\right)}}\|{\vec{x}}_{j^{\prime }}-{\vec{x}}_{j^{\prime }}^{\left(n\right)}\|{}_{2}^{2}\right),$$

where $x_{j}^{\left(n,j^{\prime }\right)}$ and $x_{j^{\prime }}^{\left(n\right)}$ are the current updated solution as a constant approximation, which is the image updated from the data-fidelity term.

Finally, the above L1 norm minimization problems can be solved with the following soft-thresholding.

(22) $$S_{\nabla }\left(x_{j}^{\left(n,j^{\prime }\right)}\right)=x_{j}^{\left(n,j^{\prime }\right)}-\{\begin{array}{c} \nabla ,\;\tau >\nabla \\ -\nabla ,\;\tau <-\nabla \\ \;\tau_{TV}\;\left(otherwise\right) \end{array},\;\;S_{\nabla }\left(x_{j^{\prime }}^{\left(n\right)}\right)=x_{j^{\prime }}^{\left(n\right)}+\{\begin{array}{c} \nabla ,\;\tau >\nabla \\ -\nabla ,\;\tau <-\nabla \\ \;\tau_{TV}\;\left(otherwise\right) \end{array},\;\left(\tau_{TV}=\left(x_{j}^{\left(n,j^{\prime }\right)}-x_{j^{\prime }}^{\left(n\right)}\right)/2,\;\nabla =\alpha^{\left(n\right)}\beta t\omega_{jj^{\prime }}\right),$$

where $S_{\nabla }\left(x_{j}^{\left(n,j^{\prime }\right)}\right)$ and $S_{\nabla }\left(x_{j^{\prime }}^{\left(n\right)}\right)$ are soft-thresholding functions. By dividing a subfunction and updating for each variable as shown in Equations (20)–(22), the solution ($S_{\nabla }\left(x_{j}^{\left(n,j^{\prime }\right)}\right),S_{\nabla }\left(x_{j^{\prime }}^{\left(n\right)}\right)$) corresponding to otherwise becomes the average of $x_{j}^{\left(n,j^{\prime }\right)}$ and $x_{j^{\prime }}^{\left(n\right)}$ ($\left(x_{j}^{\left(n,j^{\prime }\right)}+x_{j^{\prime }}^{\left(n\right)}\right)/2$). Compared to Equation (19), this weak average that occurs otherwise can reduce the error in convergence and improve the stability of convergence.

**[Update the TKV term]** We update the pixel j, $j_{k}$, $j^{\prime }$, $j_{k}^{\prime }$ simultaneously. For updating the pixel j, $j_{k}$, $j^{\prime }$, $j_{k}^{\prime }$, we further divide a subfunction $u_{g}^{TKV}\left(\vec{x}\right)$ into four subfunctions as below

(23) $$\;u^{TKV}\left(\vec{x}\right)=\frac{\beta \left(1-t\right)}{8}\overset{8}{\underset{k=1}{\sum }}\overset{G}{\underset{g=1}{\sum }}u_{g}^{TKV}\left(\vec{x}\right)=\frac{\beta \left(1-t\right)}{8}\overset{8}{\underset{k=1}{\sum }}\overset{G}{\underset{g=1}{\sum }}{\left[\omega_{jj^{\prime }}\|\left({\vec{x}}_{j}-{\vec{x}}_{j_{k}}\right)-{\left({\vec{x}}_{j^{\prime }}-{\vec{x}}_{j_{k}^{\prime }}\right)\|}_{1}^{1}\right]}_{g}\;=\frac{\beta \left(1-t\right)}{8}\overset{8}{\underset{k=1}{\sum }}\overset{G}{\underset{g=1}{\sum }}[{\left[\omega_{jj^{\prime }}{\left\|\frac{\left({\vec{x}}_{j}-{\vec{x}}_{j_{k}})-({\vec{x}}_{j^{\prime }}-{\vec{x}}_{j_{k}^{\prime }}\right)}{4}\right\|}_{1}^{1}\right]}_{x_{j}}+{\left[\omega_{jj^{\prime }}{\left\|\frac{\left({\vec{x}}_{j}-{\vec{x}}_{j_{k}})-({\vec{x}}_{j^{\prime }}-{\vec{x}}_{j_{k}^{\prime }}\right)}{4}\right\|}_{1}^{1}\right]}_{x_{j_{k}}}\;\;+{\left[\omega_{jj^{\prime }}{\left\|\frac{\left({\vec{x}}_{j}-{\vec{x}}_{j_{k}})-({\vec{x}}_{j^{\prime }}-{\vec{x}}_{j_{k}^{\prime }}\right)}{4}\right\|}_{1}^{1}\right]}_{x_{j^{\prime }}}+{\left[\omega_{jj^{\prime }}{\left\|\frac{\left({\vec{x}}_{j}-{\vec{x}}_{j_{k}})-({\vec{x}}_{j^{\prime }}-{\vec{x}}_{j_{k}^{\prime }}\right)}{4}\right\|}_{1}^{1}\right]}_{x_{j_{k}^{\prime }}}{]}_{g}\;\;=\frac{\beta \left(1-t\right)}{8}\overset{8}{\underset{k=1}{\sum }}\overset{G}{\underset{g=1}{\sum }}{\left[q\left({\vec{x}}_{j}\right)+q\left({\vec{x}}_{j_{k}}\right)+q\left({\vec{x}}_{j^{\prime }}\right)+q\left({\vec{x}}_{j_{k}^{\prime }}\right)\right]}_{g},$$

where $q\left({\vec{x}}_{j}\right)={\left[\right]}_{x_{j}}$, $q\left({\vec{x}}_{j_{k}}\right)={\left[\right]}_{x_{j_{k}}}$, $q\left({\vec{x}}_{j^{\prime }}\right)={\left[\right]}_{x_{j^{\prime }}}$, $q\left({\vec{x}}_{j_{k}^{\prime }}\right)={\left[\right]}_{x_{j_{k}^{\prime }}}$.

The proximity operator for each subfunction ($prox_{\alpha^{\left(n\right)}q}\left({\vec{x}}_{j}^{\left(n,j^{\prime },k\right)}\right)$, $prox_{\alpha^{\left(n\right)}q}\left({\vec{x}}_{j_{k}}^{\left(n\right)}\right)$, $prox_{\alpha^{\left(n\right)}q}\left({\vec{x}}_{j^{\prime }}^{\left(n,k\right)}\right)$, $prox_{\alpha^{\left(n\right)}q}\left({\vec{x}}_{j_{k}^{\prime }}^{\left(n\right)}\right)$) can be defined as follows

(24) $$\underset{{\vec{x}}_{j}}{\mathrm{argmin}}\left(\omega_{jj^{\prime }}\|{\vec{x}}_{j}-{\frac{3{\vec{x}}_{j}^{\left(n,j^{\prime },k\right)}+{\vec{x}}_{j_{k}}^{\left(n\right)}+{\vec{x}}_{j^{\prime }}^{\left(n,k\right)}-{\vec{x}}_{j_{k}^{\prime }}^{\left(n\right)}}{4}\|}_{1}^{1}+\frac{1}{2\alpha^{\left(n\right)}}\|{\vec{x}}_{j}-{\vec{x}}_{j}^{\left(n,j^{\prime },k\right)}\|{}_{2}^{2}\right),\;\underset{{\vec{x}}_{j_{k}}}{\mathrm{argmin}}\left(\omega_{jj^{\prime }}\|-{\vec{x}}_{j_{k}}+{\frac{{\vec{x}}_{j}^{\left(n,j^{\prime },k\right)}+3{\vec{x}}_{j_{k}}^{\left(n\right)}-{\vec{x}}_{j^{\prime }}^{\left(n,k\right)}+{\vec{x}}_{j_{k}^{\prime }}^{\left(n\right)}}{4}\|}_{1}^{1}+\frac{1}{2\alpha^{\left(n\right)}}\|{\vec{x}}_{j_{k}}-{\vec{x}}_{j_{k}}^{\left(n\right)}\|{}_{2}^{2}\right),\;\underset{{\vec{x}}_{j^{\prime }}}{\mathrm{argmin}}\left(\omega_{jj^{\prime }}\|-{\vec{x}}_{j^{\prime }}+{\frac{{\vec{x}}_{j}^{\left(n,j^{\prime },k\right)}-{\vec{x}}_{j_{k}}^{\left(n\right)}+3{\vec{x}}_{j^{\prime }}^{\left(n,k\right)}+{\vec{x}}_{j_{k}^{\prime }}^{\left(n\right)}}{4}\|}_{1}^{1}+\frac{1}{2\alpha^{\left(n\right)}}\|{\vec{x}}_{j^{\prime }}-{\vec{x}}_{j^{\prime }}^{\left(n,k\right)}\|{}_{2}^{2}\right),\;\underset{{\vec{x}}_{j_{k}^{\prime }}}{\mathrm{argmin}}\left(\omega_{jj^{\prime }}\|{\vec{x}}_{j_{k}^{\prime }}-{\frac{-{\vec{x}}_{j}^{\left(n,j^{\prime },k\right)}+{\vec{x}}_{j_{k}}^{\left(n\right)}+{\vec{x}}_{j^{\prime }}^{\left(n,k\right)}+3{\vec{x}}_{j_{k}^{\prime }}^{\left(n\right)}}{4}\|}_{1}^{1}+\frac{1}{2\alpha^{\left(n\right)}}\|{\vec{x}}_{j_{k}^{\prime }}-{\vec{x}}_{j_{k}^{\prime }}^{\left(n\right)}\|{}_{2}^{2}\right),$$

where ${\vec{x}}_{j}^{\left(n,j^{\prime },k\right)}$, ${\vec{x}}_{j_{k}}^{\left(n\right)}$, ${\vec{x}}_{j^{\prime }}^{\left(n,k\right)}$, ${\vec{x}}_{j_{k}^{\prime }}^{\left(n\right)}$ are the current updated solution as a constant approximation, which is the image updated from the TV term.

Finally, the above L1 norm minimization problems can be solved with the following soft-thresholding.

(25) $$S_{\nabla }\left(x_{j}^{\left(n,j^{\prime },k\right)}\right)=x_{j}^{\left(n,j^{\prime },k\right)}-\{\begin{array}{c} \nabla ,\;\tau >\nabla \\ -\nabla ,\;\tau <-\nabla \\ \;\tau_{TKV}\;\left(otherwise\right) \end{array},\;\;S_{\nabla }\left(x_{j_{k}}^{\left(n\right)}\right)=x_{j_{k}}^{\left(n\right)}+\{\begin{array}{c} \nabla ,\;\tau >\nabla \\ -\nabla ,\;\tau <-\nabla \\ \;\tau_{TKV}\;\left(otherwise\right) \end{array},\;S_{\nabla }\left(x_{j^{\prime }}^{\left(n,k\right)}\right)=x_{j^{\prime }}^{\left(n,k\right)}+\{\begin{array}{c} \nabla ,\;\tau >\nabla \\ -\nabla ,\;\tau <-\nabla \\ \;\tau_{TKV}\;\left(otherwise\right) \end{array},\;S_{\nabla }\left(x_{j_{k}^{\prime }}^{\left(n\right)}\right)=x_{j_{k}^{\prime }}^{\left(n\right)}-\{\begin{array}{c} \nabla ,\;\tau >\nabla \\ -\nabla ,\;\tau <-\nabla \\ \;\tau_{TKV}\;\left(otherwise\right) \end{array},\;\left(\tau_{TKV}=\left(x_{j}^{\left(n,j^{\prime },k\right)}-x_{j_{k}}^{\left(n\right)}-x_{j^{\prime }}^{\left(n,k\right)}+x_{j_{k}^{\prime }}^{\left(n\right)}\right)/4,\;\nabla =\alpha^{\left(n\right)}\beta \left(1-t\right)\omega_{jj^{\prime }}/8\right),$$

where $S_{\nabla }\left(x_{j}^{\left(n,j^{\prime },k\right)}\right)$, $S_{\nabla }\left(x_{j_{k}}^{\left(n\right)}\right)$, $S_{\nabla }\left(x_{j^{\prime }}^{\left(n,k\right)}\right)$, $S_{\nabla }\left(x_{j_{k}^{\prime }}^{\left(n\right)}\right)$ are soft-thresholding function.

#### 2.3.3. The Weight

In this paper, we used the weight of nonlocal means filter [25] as

(26) $$\omega_{jj^{\prime }}=\frac{exp\left(-\max\left(\|B\left(x_{j}\right)-B{\left(x_{j^{\prime }}\right)\|}_{2}^{2}-2\sigma^{2},0\right)/h^{2}\right)}{\sum_{j^{\prime }\in \Omega }exp\left(-max\left(\|B\left(x_{j}\right)-B{\left(x_{j^{\prime }})\right\|}_{2}^{2}-2\sigma^{2},0\right)/h^{2}\right)},$$

where $\|B\left(x_{j}\right)-B{\left(x_{j^{\prime }})\right\|}_{2}^{2}$ means the average Euclidean distance between patches ($B\left(x_{j}\right),B\left(x_{j^{\prime }}\right)$) centered in an interest pixel $x_{j}$ and a distant pixel $x_{j^{\prime }}$. 

**Figure 3:**
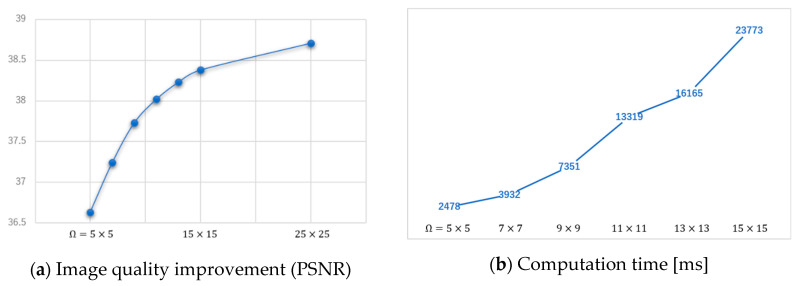
Cost performance of changing the size of weight. (**a**) Image quality improvement (PSNR), (**b**) Computation time [ms].

Theoretically, the weight must be a fixed value as a hyper-parameter of the regularization term. However, there have been previous studies showing that reweighting at each iteration contributes to better image quality [2,9]. Additionally, the larger the size of weight (Ω), the better the performance of removing artifacts or noise. As long as the computer is capable of processing, we recommend increasing the size of the weight. However, if the size of the weight is too large, the calculation cost will increase enormously as compared with the image quality improvement. Therefore, it is important to decide the size of the weight while paying attention to cost performance considering the level of artifacts or noise. Figure 3 shows the cost performance of changing the size of weight.

Next, we describe the proper estimation of the weight. If the ground truth image is known, the weight can be calculated from the ground truth image. However, in the image reconstruction, only the projection data is given and the reconstructed image and the weight must be estimated simultaneously from the projection data. Therefore, we construct the optimization by alternating the estimation of the reconstructed image and the weight.

We show the reweighting process of optimization including the data-fidelity term and regularization term:
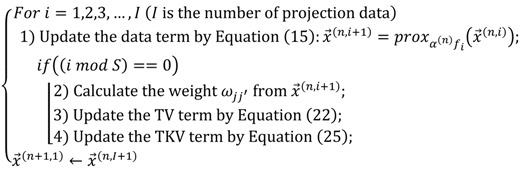
(27)
where the weight is calculated once as common to nonlocal TV and TKV and the weight is calculated from the image updated from the data-fidelity term. The span parameter S determines how often regularization is performed in the main iteration n. If S is small, many regularization updates are performed in one iteration. Theoretically, the smaller the S, the more accurate the convergence. However, since nonlocal TV has a large amount of computational complexity, it is desirable to determine an appropriate value of S.

**Figure 4:**
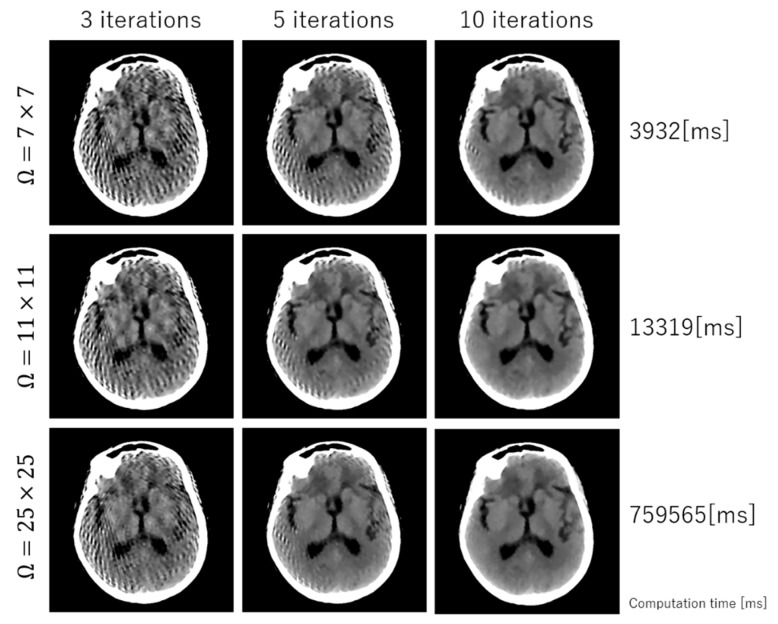
Demonstration of how the size of weight (Ω) influences image quality and computation time.

Figure 4 shows how the size of weight (Ω) influences image quality and computation time.

## 3. Experimental Results

**Figure 5:**
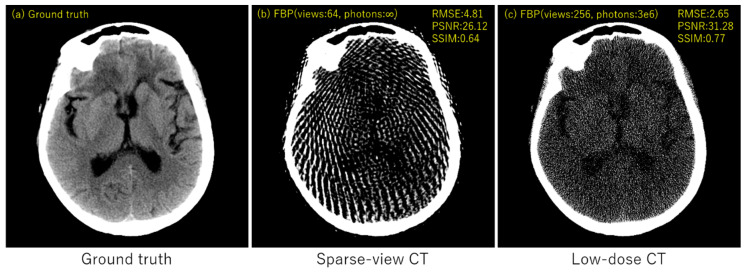
(**a**) Ground truth, (**b**) FBP with 64 projection data with no noise, (**c**) FBP with 256 projection data with the number of photon counts $3\times 10^{6}$. All images are displayed with the same window of [7.82, 62.30] HU.

**Figure 6:**
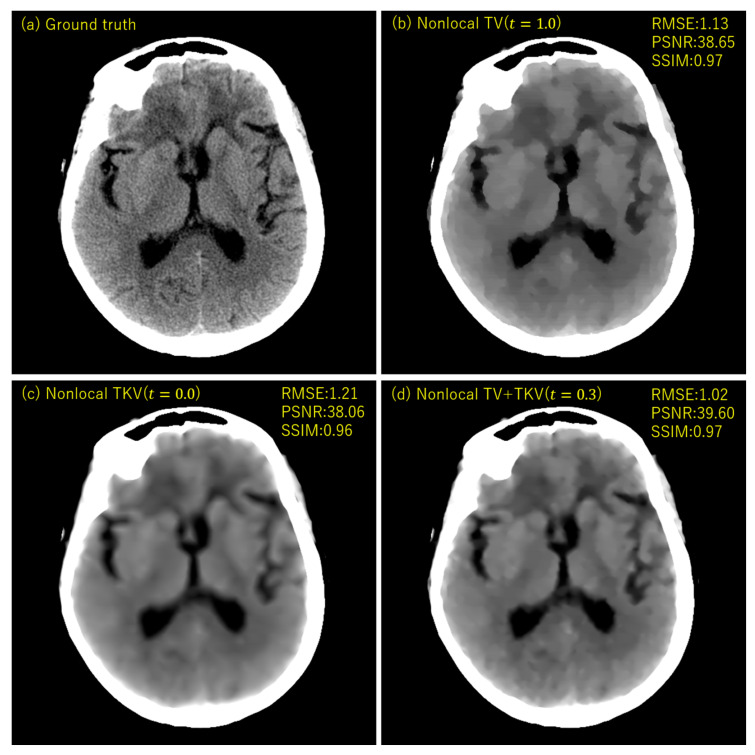
The reconstructed images of sparse-view CT (64 projection data with no noise). (**a**) Ground truth, (**b**) Nonlocal TV (t=1.0), (**c**) Nonlocal TKV (t=0.0), (**d**) Nonlocal TV+TKV (t=0.3) were compared. All images are displayed with the same window of [7.82, 62.30] HU.

**Figure 7:**
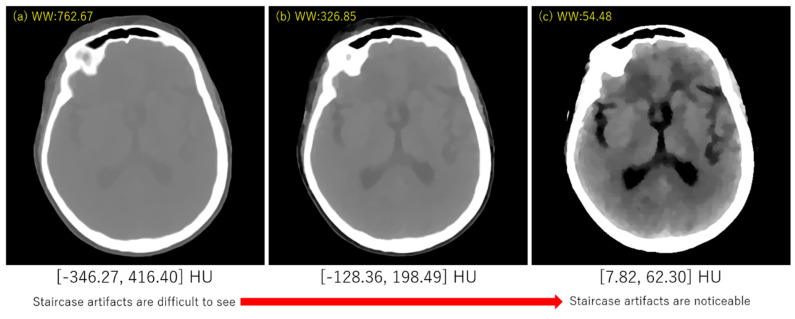
Demonstration of appearance of the staircase artifacts with various gray-scale ranges in displaying the brain CT image. (**a**) Window Width [−346.27, 416.40] HU, (**b**) Window Width [−128.36, 198.49] HU, (**c**) Window Width [7.82, 62.30] HU.

**Figure 8:**
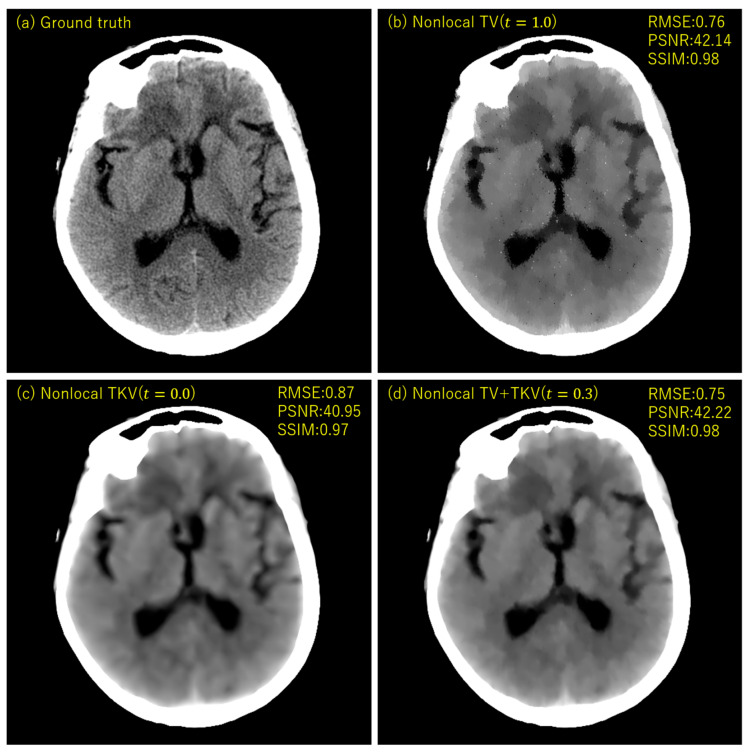
The reconstructed images of low-dose CT (256 projection data and the number of photon counts 3$\times 10^{6}$). (**a**) Ground truth, (**b**) Nonlocal TV (t=1.0), (**c**) Nonlocal TKV (t=0.0), (**d**) Nonlocal TV+TKV (t=0.3) were compared. All images are displayed with the same window of [7.82, 62.30] HU.

**Figure 9:**
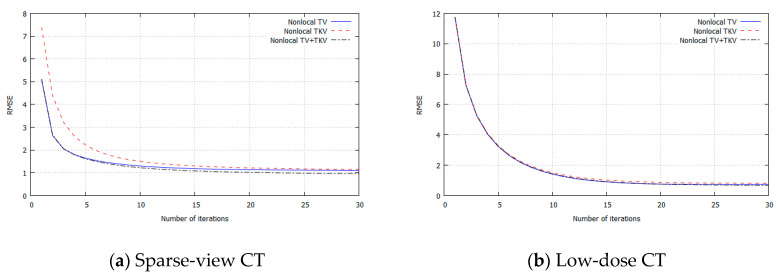
RMSE for each iteration. (**a**) Sparse-view CT, (**b**) Low-dose CT.

**Figure 10:**
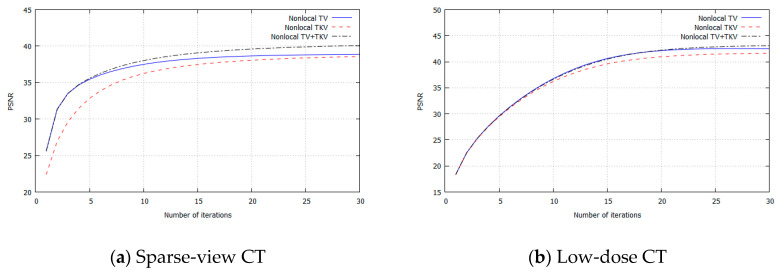
PSNR for each iteration. (**a**) Sparse-view CT, (**b**) Low-dose CT.

**Figure 11:**
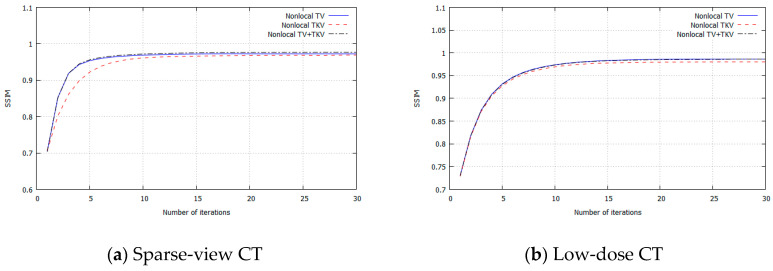
SSIM for each iteration. (**a**) Sparse-view CT, (**b**) Low-dose CT.

**Figure 12:**
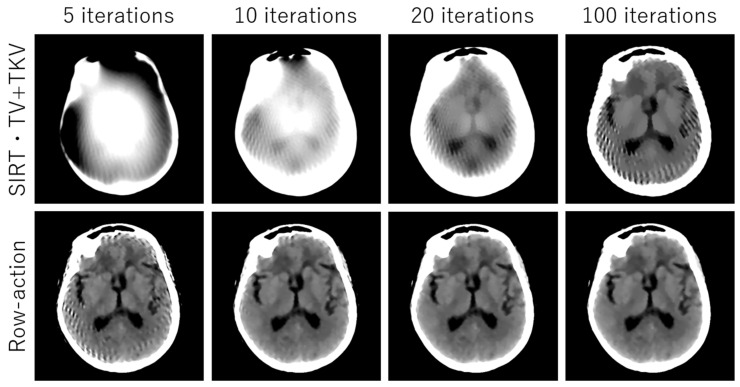
The reconstructed images of sparse-view CT (64 projection data with no noise). SIRT nonlocal TV+TKV and row-action accelerated nonlocal TV+TKV (our proposed method) were compared. All images are displayed with the same window of [7.82, 62.30] HU.

**Figure 13:**
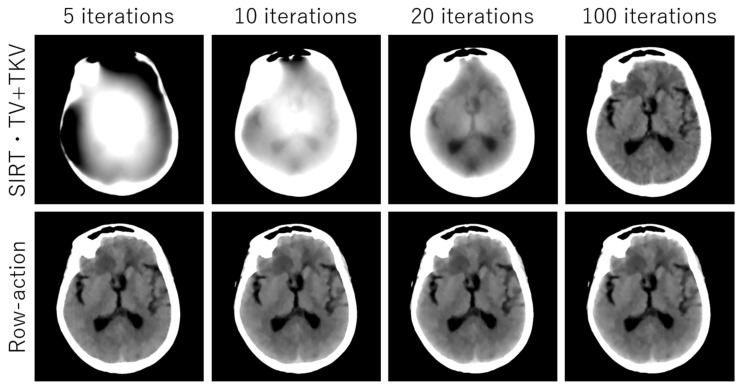
The reconstructed images of low-dose CT (256 projection data and the number of photon counts $3\times 10^{6}$). SIRT nonlocal TV+TKV and row-action accelerated nonlocal TV+TKV (our proposed method) were compared. All images are displayed with the same window of [7.82, 62.30] HU.

We performed simulation studies using a brain CT image. The reason behind using the brain image is as follows. In the brain CT imaging, the reconstructed images are shown with a compressed gray scale range much larger compared to the other CT imaging like chest imaging or abdominal CT imaging. Therefore, preserving smooth intensity changes and avoiding the staircase artifacts are much important in the brain case. Additionally, simulation studies were performed for both the sparse-view CT (the number of projection data was 64) and the low-dose CT (the number of photons was $3\times 10^{6}$). The reconstructed image consisted of 512×512 pixels, where the pixel size was 0.0585 (cm^2^). We compressed the range showing the reconstructed images to [7.82, 62.30] HU, where this contrast range was determined based on the contrast range used in clinical brain CT imaging. To evaluate image quality, standard RMSE, PSNR, SSIM values were used as metrics. The number of iterations in image reconstructions was 20 for nonlocal TV, TKV, and TV+TKV, which was determined by the fact that changes in image were small enough with this iteration number. We also showed the reconstructed images by the standard Filtered Back-Projection (FBP), and differences in image quality by changing values of the hyper-parameter t (i.e., the trade-off parameter between nonlocal TV (first derivative) term and the TKV (second derivative) term). The ground truth image and the FBP reconstructions are shown in Figure 5. The reconstructed images in the case of sparse-view CT are shown in Figure 6. In Figure 7, we show the used brain CT image with three display gray-scale ranges, from which we observe that the staircase artifacts are severe when the range of display gray-scale range is small. The reconstructed images in the case of low-dose CT are shown in Figure 8. In Figure 9, Figure 10 and Figure 11, we show convergence properties of our iterative algorithm based on Passty’s proximal splitting framework. In Figure 12 and Figure 13, to show the effect of acceleration by Passty’s proximal splitting, we incorporated the TV+TKV term into SIRT (simultaneous iterative reconstruction technique) which is a non-row-action method (a type of the standard iterative algorithm) and compared this with row-action based on our proposed nonlocal TV+TKV. From these figures, it can be observed that our algorithm converged very quickly. It is well-known that the standard iterative algorithms such as Chambolle–Pock [26] and proximal gradient algorithms require several hundreds of iteration up to the convergence. The benefit of our iterative algorithm mainly originates from the fact that our algorithm is of row-action type.

## 4. Discussion

The experimental results Table 1 showed that the reconstructed image by nonlocal TV+TKV was closest to the ground truth image, with good RMSE, PSNR and SSIM values. Furthermore, no isolated points caused by outliers of the soft-thresholding, which often appear in the TV-class reconstruction methods, were visible. In the sparse-view CT, the result of nonlocal TV (t=1.0) using only the first-order derivative was of very high-contrast, but the staircase artifacts appeared in the smooth intensity changes. In other words, the region with small intensity changes was over-smoother by the regularization as if the oil-painting. In the low-dose CT, the result of nonlocal TV showed isolated points in the region closer to the center of the image. In both the sparse-view CT and the low-dose CT, nonlocal TKV (t=0.0) using only the second-order derivative was able to preserve fine soft tissues well including textures and low-contrast objects, however, it suffered from the blurring in the edge parts. This is because, by using a large weight in the second-order derivative, the threshold value ($\tau_{TKV}$) of the soft-thresholding operation becomes very small (i.e., $\tau_{TV}\gg \tau_{TKV}$).

## 5. Conclusions

In this paper, we proposed a new concept in nonlocal TV, in which the first and second order derivatives are combined in the regularization term. By combining the two terms, we were able to compensate for each other’s weaknesses, i.e., staircase artifact and loss in smooth intensity changes in the first derivative and image blurring in the second derivative. Furthermore, we proposed a specially designed proximal splitting algorithm that is based on Passty’s framework. The key idea is to split the original cost function to minimize as finely as possible to accelerate convergence and simplify necessary computations. This allows as to make the final iterative algorithm into a form of row-action type, which is known to converge very quickly compared to other standards such as Chambolle–Pock algorithm and proximal gradient. In our experiments, we experimentally confirmed that the proposed algorithm converges within 20 iterations even for the case of brain CT imaging in which the requirement of image contrast is very severe. The simulation results with the brain CT image were performed for both the sparse-view CT and the low-dose CT. We showed that our proposed algorithm works well in practice.

As future work, our proposed nonlocal TV can be compared with the latest technology e.g., deep leaning [27,28] or other applied methods such as low-rank minimization [29,30].

Recently, image reconstruction methods using deep learning have been actively investigated. Our proposed method can be compared with existing deep leaning [27,28] as advanced compressed sensing. Additionally, our proposed method can be applied to low-rank TV, which can improve image quality by combing low-rank minimization and Total Variation [29,30].

## Figures and Tables

**Table 1:** Summary of each method.

	Nonlocal TV	Nonlocal TKV	Nonlocal TV + TKV
Convergence	**Good**	Not bad	**Good**
High contrast	**Yes**	No	**Yes**
Smooth intensity change	No	**Yes**	**Yes**
